# Long term marijuana users seeking medical cannabis in California (2001–2007): demographics, social characteristics, patterns of cannabis and other drug use of 4117 applicants

**DOI:** 10.1186/1477-7517-4-16

**Published:** 2007-11-03

**Authors:** Thomas J O'Connell, Ché B Bou-Matar

**Affiliations:** 1 Private medical practice, Oakland, CA, USA; 2 Private consultant, Mountain View, CA, USA

## Abstract

**Background:**

Cannabis (marijuana) had been used for medicinal purposes for millennia. Cannabinoid agonists are now attracting growing interest and there is also evidence that botanical cannabis is being used as self-medication for stress and anxiety as well as adjunctive therapy by the seriously ill and by patients with terminal illnesses. California became the first state to authorize medicinal use of cannabis in 1996, and it was recently estimated that between 250,000 and 350,000 Californians may now possess the physician's recommendation required to use it medically. More limited medical use has also been approved in 12 additional states and new initiatives are being considered in others. Despite that evidence of increasing public acceptance of "medical" use, a definitional problem remains and all use for any purpose is still prohibited by federal law.

**Results:**

California's 1996 initiative allowed cannabis to be recommended, not only for serious illnesses, but also "for any other illness for which marijuana provides relief," thus maximally broadening the range of allowable indications. In effect, the range of conditions now being treated with federally illegal cannabis, the modes in which it is being used, and the demographics of the population using it became potentially discoverable through the required screening of applicants. This report examines the demographic profiles and other selected characteristics of 4117 California marijuana users (62% from the Greater Bay Area) who applied for medical recommendations between late 2001 and mid 2007.

**Conclusion:**

This study yielded a somewhat unexpected profile of a hitherto hidden population of users of America's most popular illegal drug. It also raises questions about some of the basic assumptions held by both proponents and opponents of current policy.

## Methods

### Development of standardized interview

The early discovery that nearly all applicants had tried (initiated) cannabis, alcohol, and tobacco during adolescence eventually led to selection of a standardized clinical interview (SCI) as the optimum way to obtain the basic information required to assess their past use of cannabis.

Data gathered using a prototype of the SCI to screen 622 consecutive new applicants between July 1 and December 31, 2002 were analyzed in a simple relational database. Results were later reported at a May 2004 meeting and eventually published in 2005 [1]. Meanwhile, the original questions, in somewhat modified form, have been used to screen all new applicants including those seeking annual "renewals," from January 2003 on. Thus 199 of 951 (21%) of those originally screened with less searching examinations while the SCI was being developed, eventually served as their own controls. Their responses confirmed that they shared the same general characteristics as the others and also that the sensitive information sought would be provided only if specifically requested. In late 2005 a more sophisticated relational database was created and later customized with drop-down menus to allow responses to be entered directly into a laptop computer in real time, thus incorporating the database as an intrinsic part of the medical record.

### Selection of areas of interest

Once the linkage between cannabis, alcohol, and tobacco had been appreciated, questions focusing on initiation and subsequent use of all three drugs were asked of several hundred consecutive applicants. The further discovery, that many had tried other "drugs of abuse" was explored by adding questions requiring yes-no responses about their initiations of 8 specific illegal agents. When patterns in personal histories suggested that family relationships and school experiences had also played a significant role in their adolescent drug initiations, the inquiry was broadened to include those areas. A prototype of the standardized clinical interview (SCI) became ready for clinical use by July 1, 2002.

## Results

### Demographics

4117 individual applicants were seen on as many as four occasions between November 2001 and June 30, 2007. All were seeking a physicians' approval of their use of cannabis; 3187 (77.4%), were male, ranging in age from 16 to 91 when first seen (median age 31). 930 (22.6%) were female, ranging in age from 16 to 89, with a median age of 36. The median age of the entire population was 32, reflecting both the smaller number of females and their somewhat greater age when first seen.

Table 1 shows race/ethnicity for the entire population. Analysis by year-of-birth (Table 2) reveals more Asians and Hispanics among the younger applicants, reflecting the two groups that have been immigrating to California in the greatest numbers in recent years. Analysis by both age and race also revealed other differences.

**Table 1:** Race/ethnicity of entire population (N = 3515). As subsequently shown by a more searching analysis, the composition of the applicant population has been changing steadily.

**Caucasian**	68.8%
**African American**	16.2%
**Hispanic**	8.1%
**Asian**	5.1%
**Other**	1.7%

**Table 2:** Cohort analysis of race/ethnicity (N = 3185). Analysis of racial composition by year of birth cohorts also shows that the applicant population has reflected immigration trends.

	**1936–1945**	**1946–1955**	**1956–1965**	**1966–1975**	**1976–1985**
**Caucasian**	68.3%	76.6%	74.0%	65.4%	66.3%
**African American**	24.2%	17.5%	16.9%	19.0%	12.7%
**Hispanic**	5.0%	3.5%	4.8%	7.6%	11.6%
**Asian**	0.8%	1.0%	3.0%	5.5%	8.0%
**Other**	1.7%	1.4%	1.3%	2.6%	1.4%

Tables 3 and 4 summarize educational and occupational histories; Table 5 provides data on applicants who were unemployed when first seen. Overall, this population exhibited lower High School drop out rates and higher percentage of graduates than national averages. The percentages earning Bachelors' degrees and Doctorates are nearly identical to the national average, but only about one half as many had earned Masters' degrees.

**Table 3:** Highest Education Attainment over 25, Applicants compared to US Population (N = 936). In general, cannabis applicants compared favourably with national averages.

	**Patient**	**US**
**Drop Out**	11.1%	14.1%
**Diploma**	61.3%	49.0%
**Associate**	5.3%	8.8%
**Bachelors**	18.3%	18.4%
**Master/Prof**	2.7%	8.4%
**Doctorate**	1.3%	1.3%

**Table 4:** Occupational divisions for employment for applicants and US population (N = 2092). The two groups are quite similar with the exception of Construction and Extraction, Office and Administrative Support, which are gender specific professions.

**Occupational Divisions**	**Patient**	**US**
Management	4.59%	4.57%
Business and Financial Operations	3.25%	4.15%
Computer and Mathematical	3.59%	2.27%
Architecture and Engineering	1.72%	1.83%
Life, Physical, and Social Science	.53%	.91%
Community and Social Service	1.67%	1.3%
Legal	.76%	.76%
Education, Training and Library	3.15%	.62%
Arts, Design, Entertainment Sports and Media	7.46%	1.29%
Healthcare Practitioner and Technical	1.58%	5.02%
Healthcare Support	2.82%	2.58%
Protective Service	1.34%	2.35%
Food Preparation and Service Related	6.98%	8.29%
Building and Grounds Cleaning and Maintenance	2.63%	3.33%
Personal Care and Service	2.15%	2.45%
Sales and Related	9.03%	10.69%
Office and Administrative Support	3.35%	17.49%
Farming, Fishing and Forestry	.72%	.34%
Construction and Extraction	18.36%	4.89%
Installation, Maintenance And Repair	8.56%	4.07%
Production	10.9%	7.87%
Transportation and Materials Moving	4.88%	7.36%

**Table 5:** Non-occupational divisions for applicants and US population (N = 494) The two groups are quite similar except for the relative scarcity of retirees in the applicant population.

**Non-Occupational Divisions**	**Patient**	**US**
Student	8.62%	8.86%
Disabled	3.56%	4.1%
Retired	3.44%	16.92%
Unemployed	3.48%	3.33%

Their occupations resembled US averages in some employment areas and were quite different in others (Table 4); in terms of non-occupational divisions (Table 5), a much smaller percentage are retirees, a finding that reflects both their relative youth and the paucity of applicants born before 1946.

Although the extremes of applicant age ranged from 16 to 91, only 3 were under 18 when first seen. The great majority (84.16%) were between 21 and 60, a finding further emphasized when the population is examined by year of birth (Table 6), a perspective that also discloses how few (4.53%) had been born before 1946. The overall male female ratio was nearly four to one (Table 7); however when examined as year of birth cohorts, it varies from over 5:1 for the youngest applicants to almost 3:1 for the oldest. Nearly 70% were Caucasians and 16% were Black, with sizable numbers of Hispanics and Asians (Table 1).

**Table 6:** Distribution by year of birth cohorts (N = 3946). This further emphasizes that one's birth cohort determines what drugs one can try during adolescence.

**Before 1945**	4.5%
**1946–1955**	14.6%
**1956–1965**	17.3%
**1966–1975**	25.9%
**1976–1985**	35.1%
**After 1986**	2.6%

**Table 7:** Birth cohorts and gender (N = 3906). Although women were outnumbered by men in each cohort, there were significant differences noted with age.

	**Male**	**Female**
**1936–1945**	73.4%	26.6%
**1946–1955**	68.6%	31.4%
**1956–1965**	72.5%	27.5%
**1966–1975**	80.1%	19.9%
**1976–1985**	82.5%	17.5%
**≥ 1986**	82.3%	17.7%

### Initiation and use of cannabis

An overwhelming majority (87.9%) of 3038 applicants queried about the details of their cannabis initiation had tried it before the age of 19, usually in the company of older siblings, cousins or peers. After subtracting those born before 1946, the percentage of applicants who had tried marijuana before the age of twenty went up to 90%. Some became regular users almost immediately, while others remained sporadic users for years (that interval was estimated by asking them when they first began to "buy their own").

### Amounts and patterns of cannabis use

Essentially all applicants queried about their current use were consuming inhaled cannabis on a regular basis in amounts that varied considerably, but tended to remain stable over time. The range is from less than one sixteenth ounce per week to over one ounce, with about 70% estimating they consume between 1/8 and 1/4 oz./week. Almost 90% acknowledge daily, or near daily ("six days a week") use, with about 10% insisting their use is far less frequent, in the range of two to five days/week.

### Mode of cannabis use

There was a decided preference for inhaled cannabis. Most had not tried edibles until their own recommendation, or that of a friend, gave them access to edibles from a club or dispensary. Only 50 of 830 (6%) questioned about edibles were using them on a regular basis. The reasons given were that edible effects were more difficult to control and more likely to be undesirable and/or prolonged.

### Initiation and use of tobacco and alcohol

**Figure 1:**
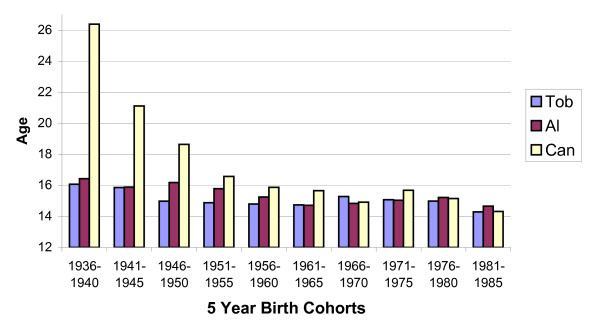
**Average initiation age tobacco, alcohol and cannabis**. Those born before 1940 were fewest in number; they had also tried cannabis at the oldest average age. Baby Boomers born after 1946 were the first large cohort, and their successors were still younger when they tried cannabis. The 61–65 cohort initiated cannabis, alcohol, and tobacco at essentially the same average age.

One of the more significant patterns revealed by comparing average initiation ages for cannabis, alcohol and tobacco within the context of birth cohorts was that the oldest Baby Boomers had tried cannabis at a considerably later age than their younger successors. By 1975, less than ten years after the "Summer of Love," in 1967, cannabis was being initiated by over half of all American adolescents at close to the same average ages they also were trying alcohol and tobacco (Table 8, Figure 1).

**Table 8:** Average initiation ages for entry level agents (N = 2498). This table is depicted by Figure 1 and emphasizes the rapid fall in age at initiation of cannabis after it first became available in high schools.

	**Tobacco**	**Alcohol**	**Cannabis**
**1936–1940**	16.07	16.43	26.39
**1941–1945**	15.86	15.89	21.12
**1946–1950**	14.98	16.18	18.64
**1951–1955**	14.88	15.79	16.58
**1956–1960**	14.8	15.25	15.87
**1961–1965**	14.74	14.71	15.66
**1966–1970**	15.28	14.84	14.92
**1971–1975**	15.08	15.04	15.68
**1976–1980**	14.99	15.22	15.15
**1981–1985**	14.29	14.66	14.32

Essentially all applicants also admitted to trying alcohol. Nearly two thirds (64.3%) of the 1226 specifically queried about alcohol blackouts had experienced at least one and 6.26% admitted to four or more. Of 1214 applicants asked to compare their current alcohol consumption with their previous lifetime peak, 130 (10.7%) claimed to be abstinent, 341 (28%) said they were drinking less than 5% of their lifetime peaks, and an overwhelming 1058 (87%) claimed to be drinking less than half as much. Most of those who noted little change from their lifetime peaks had been moderate drinkers to begin with. This is evidence that once cannabis was established as their drug of choice, this population's subsequent alcohol consumption diminished; both collectively, and as individuals, a finding that clearly deserves further evaluation.

A history of cigarette initiation, later followed by chronic use, was prevalent in this population. 2559 of 2741 (96.4%) applicants, when asked if they had ever tried inhaling a cigarette, had done so; of 1324 who were specifically queried about their lifetime cigarette use, 872 (65.8%) had become daily smokers for some length of time. Although all but four of those still smoking claim they want to quit, only 316 (36.2%) of all smokers (23.9% of respondents) had been able to do so by the time of the interview. Most who are still smoking have reduced their daily cigarette consumption; a majority relate temporary increases in their daily cigarette use to "stress." Thus the impact of daily cannabis use on cigarette consumption, although less impressive than is the case with alcohol, also seems significant and worthy of further exploration.

### Other drug initiations

**Figure 2:**
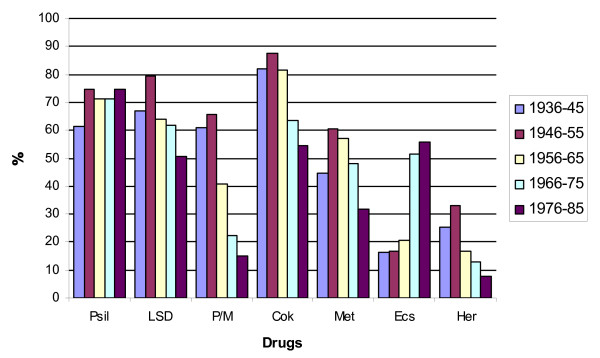
**Other illegal drugs tried by 10 year cohort analysis**. Interestingly, while all cohorts sampled other illegal drugs aggressively during adolescence, the rates at which they've done so have fallen progressively. Note also the striking generational differences in peyote/mescaline initiations by older cohorts and ecstasy by younger ones.

When examined from the standpoint of both year of birth (YOB) cohorts and admitted initiations of other illegal agents (Table 9, Figure 2) noticeable and consistent differences are revealed: whites in every age cohort had consistently tried all other illegal agents more frequently than other racial groups (Table 10).

**Table 9:** Initiation rates for other illegal drugs by YOB cohorts (N = 2364). With the exception of "magic mushrooms," and ecstasy (a psychedelic made illegal in 1988), initiation rates for all Schedule One drugs have declined since 1975.

	**1936–45**	**1946–55**	**1956–65**	**1966–75**	**1976–85**
**Psilocybin**	61.36%	74.52%	71.06%	71.07%	74.58%
**LSD**	67.05%	79.56%	63.79%	61.68%	50.60%
**P/M**	60.92%	65.75%	40.89%	22.27%	15.09%
**Cocaine**	81.82%	87.60%	81.50%	63.59%	54.40%
**Meth**	44.83%	60.44%	57.21%	48.28%	31.56%
**MDMA**	16.28%	16.57%	20.79%	51.49%	55.89%
**Heroin**	25.29%	33.06%	16.67%	13.02%	7.69%

**Table 10:** Initiations of other illegal drugs by race (N=2400). Although race seems related to initiation rates throughout, this shows that drug initiations by all aces trying cannabis have been falling proportionately as the adolescent market matured.

	**Caucasian**	**African American**	**Hispanic**	**Asian**
**Psilocybin**	82.33%	41.08%	53.50%	65.21%
**LSD**	69.56%	28.13%	43.71%	42.47%
**P/M**	35.19%	21.42%	15.34%	19.82%
**Coke**	74.21%	49.76%	55.55%	51.30%
**Meth**	52.59%	20.28%	34.67%	30.08%
**MDMA**	43.43%	28.88%	31.21%	64.65%
**Heroin**	17.54%	10.50%	7.10%	7.75%

Further cohort analysis of this population's adolescent interest in other illegal drugs, plus its nearly universal initiation of alcohol and tobacco, suggest that while race (Table 10), and generation (Table 9) exert significant influences, gender merely parallels ethnicity (Table 11).

**Table 11:** Initiations of Other Illegal Drugs by Gender (N=2464). Similarly, although women consistently tried all agents somewhat less often than men, the close parallels and internal consistency suggests the data are reliable.

	**Male**	**Female**
**Psilocybin**	74.31%	62.41%
**LSD**	60.06%	57.54%
**P/M**	30.93%	28.17%
**Coke**	67.32%	65.85%
**Meth**	44.82%	44.01%
**MDMA**	41.61%	37.57%
**Heroin**	15.86%	12.32%

Despite such differences (Tables 9 &10), all cohorts and racial groups have shown steady downward trends in their initiation of all other illegal drugs, with the interesting exception of psychedelic mushrooms (psilocybin) and, perhaps, ecstasy (MDMA).

## Discussion

It has long been recognized that users of illegal drugs may be difficult to identify, let alone recruit into a study [2]. That chronic users of cannabis would seek medical evaluations and be so willing to share sensitive personal information within the context of their required evaluations was the unanticipated benefit of Proposition 215 that made this study possible.

Birth cohort analysis of the average ages at which applicants reported first trying alcohol, tobacco and cannabis (Table 8, Figure 1) demonstrates that a surge in youthful marijuana use began in the US in the mid Sixties. However, that event was not documented until publication of the first Monitoring the Future (MTF) data in 1975 demonstrated that over half of American adolescents were trying marijuana while still in High School [3].

Close questioning of applicants suggests that the majority had been motivated by a mix of physical and emotional symptoms which had been experienced at varying times in their lives. Further, that a majority had become initiates, and later chronic users of cannabis under circumstances that suggest that it was for relief of emotional symptoms in most instances. Their discovery (usually later), that cannabis also relieved physical symptoms, was most frequently made within a context of established chronic use. That notion is further supported by recent literature indicating that phytocannabinoids, newly discovered endocannabinoids, and synthetic cannabinoid agonists all seem to manifest anxiolytic effects in both humans and animals [4-8].

More than 85% of applicants had tried other illegal drugs, principally lysergic acid diethylamide (LSD), psilocybin, cocaine, and/or MDMA. The majority of those doing so hadn't remained chronic users of any except cannabis. While a majority have continued to use alcohol occasionally, the volumes consumed and the occurrence of events related to alcohol excess have sharply diminished.

A "gateway" hypothesis had developed from observations [9] that most marijuana users studied in the early Seventies were adolescents and young adults who had first tried alcohol and tobacco; also that many had tried marijuana before later trying heroin. However, subsequent efforts to establish a definitive causal link between marijuana and "harder" drugs have been largely unsuccessful [10]. More recently, a theoretical alternative was shown to provide an explanation for accumulated MTF data that is at least as coherent [11].

A significant percentage of male applicants under 30 had been treated or evaluated for treatment with Ritalin or other stimulants for attention deficit hyperactivity disorder (ADHD) as children and their histories of a preference for morning use of minimal amounts strongly suggest that inhaled cannabis enhances their ability to concentrate. The statement of one, a construction company estimator, was revealing: "after two hits (of marijuana), and my morning coffee I'm the best estimator in the company." Another, a dental technician, stated that, when I first look at my workbench, I think I'll never finish, but after a couple of tokes (of marijuana), I'm through (with work) by two o'clock." Thus, reduction of work related anxiety seems a major factor in deciding to apply for legalized use of cannabis.

## Conclusion

Analysis of the demographic and social characteristics of a large sample of applicants seeking approval to use marijuana medically in California supports an interpretation of long term non problematic use by many who had first tried it as adolescents, and then either continued to use it or later resumed its use as adults. In general, they have used it at modest levels and in consistent patterns which- anecdotally- often assisted their educational achievement, employment performance, and establishment of a more stable life-style. These data suggest that rather than acting as a gateway to other drugs, (which many had also tried), cannabis has been exerting a beneficial influence on most.

Anecdotal evidence from repeated clinical contacts, and other data gathered incidentally over five years of experience with this population suggests that, except for very modest alcohol consumption and obligatory (addictive) use of tobacco by those trying to quit, cannabis is the only drug used past the age of twenty-five by most. Indeed, their total drug use histories suggest that by competing successfully with other, potentially more harmful agents, cannabis may have actually been protective. Evidence from federal agencies confirms that, since 1970, there has been a gradual decrease in consumption of both tobacco and alcohol (with correlated improvements in health outcomes) even as cannabis initiation by adolescents has remained at significant levels and overall chronic use by adults has been rising steadily.

While this is a self-selected sample (which restricts the generalizations that can be made from the observations reported), its large size, the consistency of the patterns uncovered, as well as their alcohol and tobacco outcomes, seem significant. For the majority, cannabis can be seen as an effective anxiolytic/antidepressant, performing as well or better than many currently available pharmaceutical agents prescribed for the same symptoms. This finding lends important support to the concept of allowing cannabis to be used medically by all those who have been chronic users and found it beneficial.

## Abbreviations

Attention deficit hyperactivity disorder (ADHD)

Cannabis (Marijuana)

Cocaine (Coke)

Ecstasy (MDMA)

Lysergic acid diethylamide (LSD)

Monitoring the Future (MTF)

Peyote/mescaline (P/M)

Psychedelic mushrooms (Psilocybin)

Standardized clinical interview (SCI)

Tokes (Marijuana)

Year of birth (YOB)

## Authors' contributions

TJO conceived the study, designed it, conducted all the clinical interviews, and wrote the report.

CBB designed the relational data-base for data analysis and later modified it to serve as medical record since December 2005. Conducted statistical analysis of data and contributed several other valuable suggestions and helped write and edit the report.
